# Visualization of quantized vortex reconnection enabled by laser ablation

**DOI:** 10.1126/sciadv.abn1143

**Published:** 2022-05-04

**Authors:** Yosuke Minowa, Shota Aoyagi, Sosuke Inui, Tomo Nakagawa, Gamu Asaka, Makoto Tsubota, Masaaki Ashida

**Affiliations:** 1 Graduate School of Engineering Science, Osaka University, 1-3, Machikane-yama, Toyonaka, Osaka, Japan.; 2 JST, PRESTO, 4-1-8 Honcho, Kawaguchi, Saitama, Japan.; 3 Department of Physics, Osaka City University, 3-3-138 Sugimoto, Osaka, Japan.; 4 Nambu Yoichiro Institute of Theoretical and Experimental Physics (NITEP), Osaka City University, 3-3-138 Sugimoto, Osaka, Japan.; 5 The Advanced Research Institute for Natural Science and Technology (OCARINA), Osaka City University, 3-3-138 Sugimoto, Osaka, Japan.; 6 Department of Physics, Osaka Metropolitan University, 3-3-138 Sugimoto, Osaka, Japan.

## Abstract

Impurity injection into superfluid helium is a simple and appealing method with diverse applications, including high-precision spectroscopy, quantum computing with surface electrons, nano/micromaterial synthesis, and flow visualization. Quantized vortices play a major role in the interaction between superfluid helium and light impurities. However, the basic principle governing this interaction is still unclear for dense (high mass density and refractive index) materials, such as semiconductor and metal impurities. Here, we provide experimental evidence of the dense silicon nanoparticle attraction to the quantized vortex cores. We prepared the silicon nanoparticles via in situ laser ablation. Following laser ablation, we observed that the silicon nanoparticles formed curved filament–like structures, indicative of quantized vortex cores. We also observed that two accidentally intersecting quantized vortices exchanged their parts, a phenomenon called quantized vortex reconnection. This behavior closely matches the dynamical scaling of reconnections. Our results provide a previously unexplored method for visualizing and studying impurity-quantized vortex interactions.

## Teaser

Semiconductor nanoparticles prepared in situ via laser ablation decorate quantized vortices in superfluid helium.

## INTRODUCTION

Bose-Einstein condensation is a remarkable manifestation of macroscopic quantum coherence, which does not entail any classical analog and has been the focus of intensive studies on fundamental quantum mechanics. Among condensate types, superfluid ^4^He has a relatively higher transition temperature, allowing us to prepare a substantially larger number of superfluid atoms (*N* ∼ 10^25^). Thus, superfluid ^4^He serves as an eminent platform for studying the interaction between condensates and impurities, including atoms (*1*), ions (*2*), electrons (*3*), surface electrons (*4*), and frozen hydrogen, deuterium, and air particles (*5*–*7*), because the introduced impurities immediately come into thermal equilibrium with the surrounding condensates without destroying the superfluidity. A remarkable example is the interaction between a quantized vortex and light impurities such as electron bubbles (*3*). A quantized vortex is a stable topological defect that represents the macroscopic quantum nature of the superfluid ^4^He. Quantized vortex physics facilitates the understanding of the fundamental properties of superfluid ^4^He. Because of a Bernoulli pressure gradient, electron bubbles are attracted to the quantized vortex core, where they are localized. The trapped electron bubbles have been used to visualize the quantized vortex lattice (*3*). Similar interactions have been extensively studied for other light impurities (low mass density, low refractive index, *n* ∼ 1). A stunning example is the visualization of quantized vortex dynamics using frozen hydrogen particles (*5*, *8*). However, although some reports have indicated that dense materials, such as metallic or semiconducting nanowires, are formed along the quantized vortex core (*9*–*12*), the quantized vortex contribution remains unclear. Some reports have suggested that normal fluid eddies can also attract metallic impurities (*10*, *13*). Here, we provide direct experimental evidence of dense silicon nanoparticle attraction to the quantized vortex and the localization along the vortex core. The density (ρ_Si_ ∼ 2400 kg/m^3^) and the refractive index (*n*_Si_ = 4.14 here) of silicon nanoparticles are distinctly larger than those of liquid helium (ρ_He_ ∼ 145 kg/m^3^, *n*_He_ = 1.028). The optical refractive index roughly correlates with the mass density (*14*). The injection of dense impurities into the superfluid ^4^He itself is not a straightforward task owing to the cryogenic environment. This is because impurities are prone to stick to the substrate and aggregate, making it difficult to prepare isolated impurities in the superfluid ^4^He. We overcame this problem using a laser ablation technique, which allows the silicon nanoparticles to be prepared in the superfluid ^4^He. Laser ablation, or sputtering, is a unique in situ preparation method to produce atoms, ions, and nano/microparticles in various environments; it is also applicable to superfluid ^4^He. Laser ablation in superfluid ^4^He has been used for precision spectroscopy (*1*, *15*, *16*), tracer injection (*17*), nano/microstructure formation (*10*, *18*), and optical manipulation/trapping (*19*, *20*). In this study, we observed the silicon nanoparticle–decorated, suspended quantized vortices, and identified the vortex reconnection events (*21*), whose dynamics were found to be consistent with dimensional analysis results and simple vortex filament model (VFM) calculations.

## RESULTS

### Nanoparticle synthesis via laser ablation and suspended quantized vortex observation

**Fig. 1.**
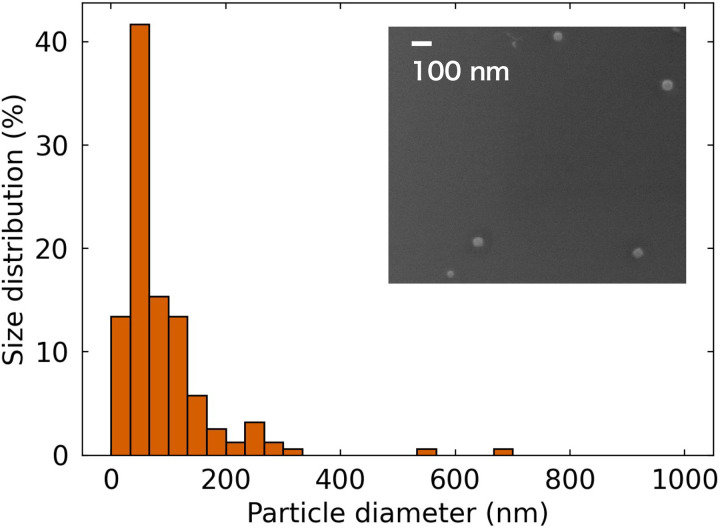
Silicon nanoparticles fabricated via laser ablation in superfluid ^4^He. Silicon nanoparticle size distribution. Inset: SEM image of typical silicon nanoparticles.

**Fig. 2.**
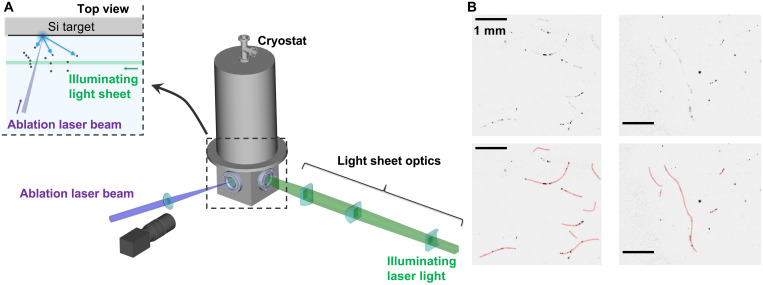
Observation of suspended quantized vortices decorated with silicon nanoparticles in superfluid ^4^He. (**A**) Experimental setup schematics. Ablation laser pulses enter the cryostat from the front window and irradiate the silicon target. The nanoparticles localized along the quantized vortex core are illuminated from the side window with a light sheet. The scattered light is imaged through the front window onto a high-speed complementary metal-oxide semiconductor (CMOS) camera. Inset: Schematic top view of the cryostat interior. Nanoparticles become dispersed after the laser ablation process. (**B**) Negative images of suspended quantized vortices visualized with silicon nanoparticles (see also movies S1 and S2). Red lines indicate silicon nanoparticle structures (bottom). Scale bars, 1 mm.

We prepared semiconductor silicon nanoparticles via laser ablation at 1.4 K. A single crystalline silicon target was placed in superfluid ^4^He and was irradiated with a laser light pulse. Subsequently, target melting and evaporation and atom/ion/cluster ejection were initiated. Then, nanoparticles were observed following abrupt cooling of the ejected materials. The inset in Fig. 1 shows a scanning electron microscopy (SEM) image of the produced silicon nanoparticles observed at room temperature. The measured size distribution revealed that 70% of the particles are smaller than 100 nm. We observed the dynamics of the dispersed silicon nanoparticles under the illumination of a light sheet, as shown in Fig. 2A (see Materials and Methods for details). Figure 2B (top) shows typical negative (intensity inverted) images of the silicon nanoparticles suspended in superfluid ^4^He. Note the small nanoparticle sizes relative to the spatial resolution of the applied optical system. Thus, the apparent size of the bright area should be interpreted as the scattering strength of the illuminating light rather than the actual particle size. Many silicon nanoparticles were arranged in curved filaments that moved without disrupting the filament-like arrangement (see also movies S1 and S2). The images in Fig. 2B (bottom) were modified to enable better visualization of the filament-like structure locations.

### Quantized vortex reconnection observation

**Fig. 3.**
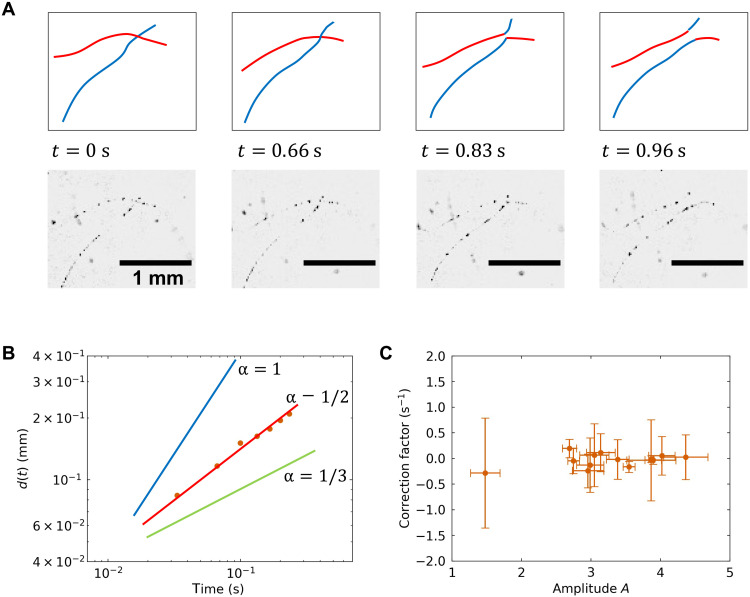
Reconnecting quantized vortices. (**A**) Bottom: Image sequence of reconnecting quantized vortices, as captured by a high-speed CMOS camera. Top: Corresponding schematics of the reconnection. The moment of reconnection was *t*_0_ = 0.79 s. (**B**) Intervortex distance as a function of the time after the reconnection. Solid lines represent the power law, *d*(*t*) ∝ (*t* − *t*_0_)^α^ for different α values. (**C**) Distribution of fitted parameters: dimensionless amplitude *A* and correction factor *c*. Each error bar shows the SD resulting from the fitting.

The shape of the observed filament-like structures indicated that the loaded silicon nanoparticles were localized along the quantized vortex cores. Furthermore, we found filament reconnection events, which are one of the most characteristic phenomena of the quantized vortices (*8*, *22*). When the two silicon nanoparticle–decorated filaments intersected, there was an exchange, as shown in the bottom row of Fig. 3A and movie S3. The corresponding schematic sequence is also shown in the top row of Fig. 3A. Upon intersecting, the two filaments abruptly moved apart. The velocity and acceleration were clearly distinct from the slow background flow. These results demonstrate that this intersection and subsequent evolution are consistent with the theoretically predicted quantized vortex motion known as quantized vortex reconnection (*21*). If we assume that there was no characteristic length scale involved in the reconnection event, then the intervortex distance *d* (or any physical quantity having length dimension) after reconnection can be expected to follow the dynamical scaling given below

$$d(t)=A\sqrt{\kappa (t-t_{0})}$$ (1)

where *A* is a dimensionless amplitude factor and *t*_0_ is the moment of reconnection. This dynamical scaling has been previously observed (*7*). Equation 1 was deduced via dimensional analysis. The length dimension is only included in the circulation quanta κ = *h*/*m*, where *h* is the Planck constant and *m* is the mass of a helium atom. Thus, the intervortex distance temporal evolution should be written as Eq. 1. Figure 3B depicts the intervortex distance measured for a pair of nearest nanoparticles in two reconnecting vortices after the moment of reconnection shown in Fig. 3A. Figure 3B also displays three different lines corresponding to the power law *d*(*t*) ∝ (*t* − *t*_0_)^α^ for different α values. The close match between the experimental data and power law *d*(*t*) ∝ (*t* − *t*_0_)^0.5^ indicates that the observed phenomenon is indeed the manifestation of the quantized vortex reconnection (*23*, *24*).

To further elucidate on the nature of the quantized vortex reconnection, we collected a number of similar events as follows. Because of the wide depth of field of our applied optical system, the three-dimensional quantized vortex motion was projected onto a two-dimensional image. To prevent this from leading to an underestimation of the intervortex distance, we illuminated the suspending quantized vortices with a light sheet (see Materials and Methods for details). Then, the three-dimensional arrangement of the reconnecting vortices was only partly visualized. We focused on events wherein one of the reconnecting vortices was decorated and visualized with only one silicon nanoparticle. During the reconnection event, the nanoparticle collided with the visualized segment of the other quantized vortex; then, the magnitude and direction of the nanoparticle velocity suddenly changed, indicating the moment of reconnection and following the repelling motion of the vortices. The measured intervortex distance can be fitted with the following equation

$$d(t)=A\sqrt{\kappa (t-t_{0})}(1+c(t-t_{0}))$$ (2)

where *c* is a correction factor. The correction considers localized environmental effects such as neighboring vortices and boundary conditions (*25*). The resulting fitted parameter distribution is shown in Fig. 3C. As shown, the correction factors clustered around *c* = 0, demonstrating consistency with the dimensional argument prediction. The dimensionless amplitude factor *A* mainly spanned from 2.5 to 4.5. Because the dimensional analysis did not provide any information regarding the value of *A*, we conducted a numerical simulation using a VFM.

### VFM calculations

**Fig. 4.**
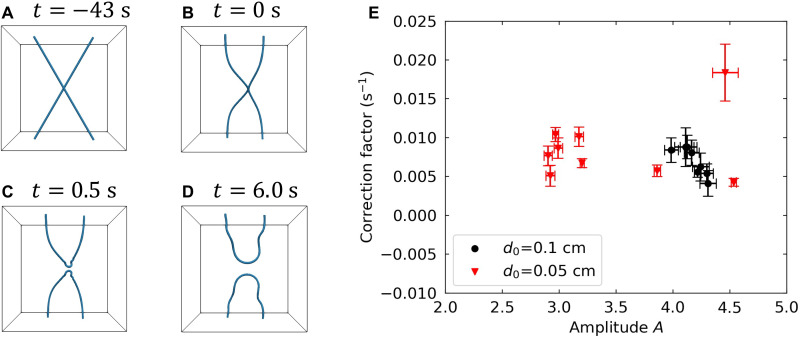
Reconnecting quantized vortex filament calculation. (**A** to **D**) Snapshots of simulated quantized vortex filament reconnection. Each cube length was 1.0 cm, and the top and bottom surfaces were subject to a solid boundary condition. (**E**) Distribution of fitted parameters: dimensionless amplitude *A* and correction factor *c* for the simulated results. Each scattered data point corresponds to a single set of simulation with some initial angle θ ∈ [0, π/2], and each error bar shows the SD resulting from applying least squares fitting to the numerical results of Eq. 2.

Several numerical studies have reproduced the dynamics of quantized vortices in superfluid ^4^He. One of the most successful approaches has been to treat a vortex line as a filament with an infinitesimal core size; this approach is known as the VFM (see Materials and Methods for more details). Although the Gross-Pitaevskii model (*26*), another successful approach, provides a microscopic description, the VFM is well suited to investigate vortex dynamics in a large volume, which is comparable to the experimental conditions used in this study. In the numerical simulations, we placed a pair of straight vortex lines in a 1.0-cm-long cubic box at *T* = 1.4 K. To properly reproduce the actual experimental setup, we set the initial vortex configuration as follows: The vortices were initially separated by *d*_0_ = 0.1 and 0.05 cm at their closest points and skewed to each other by some angle θ (θ = 0 for antiparallel and π for parallel alignments; see Fig. 4, A to D, for the numerical simulation with *d*_0_ = 0.1 cm and $\theta =\frac{\pi }{3}$). By varying θ, we numerically estimated the closest distance *d* as a function of time *t* to verify the relation shown in Eq. 2. Figure 4E shows that the correction factor *c* was relatively small for all amplitudes; this result is consistent with the experimental results shown in Fig. 3C. The distribution of *A* implies that it is dependent on the initial vortex configuration, i.e., θ and *d*_0_. In the experiment, the actual vortex configuration cannot be controlled before the reconnection event taking place, so we can think of it as somewhat randomly distributed. The numerically obtained range of *A* was 2.5 to 4.5, which is also in good agreement with the experimental results and the previous reports (*26*–*28*). Thus, we can conclude that the characteristic motions of the aggregated silicon particles indeed stems from the reconnection events of a pair of quantized vortices.

## DISCUSSION

In this study, we demonstrated that the dense silicon nanoparticles are localized along the quantized vortex cores and move collectively with the quantized vortex. The observed quantized vortices may arise from the spontaneous formation through the superfluid phase transition (*29*) or may be generated from rapid and random flows initiated by the pulsed laser ablation process. We conducted the same experiment in liquid helium in its normal phase above the superfluid transition temperature at 2.5 K and observed no filament-like structures confirming the role of quantized vortices. This finding clearly supports the hypothesis that the quantized vortices play a major role in the semiconducting or metallic nanowire formation in superfluid ^4^He (*9*, *10*, *12*, *13*, *30*–*32*), although the intermediate process needs to be further studied. Because of the Bernoulli pressure gradient around the vortex lines, the nanoparticles are thought to be attracted toward the vortex cores irrespective of their mass density (*33*). The attracting force is also derived from the inertial term in the equation of the motion (see the Supplementary Materials). The nanoparticle separation between neighboring nanoparticles along the vortex cores seems to be stable most of the time for short time scales. The separation mechanism is unclear and needs to be further experimentally and theoretically studied. The laser ablation method can inject various nanoparticles, ranging from semiconductors (*19*) to metals (*10*, *20*), into superfluid ^4^He. This material diversity feature is important for the integration of quantized vortex research and optical probing and manipulation because it enables the selection of an appropriate material based on favorable optical properties. The refractive index of silicon (*n*_Si_ = 4.14) is indeed much larger than that of frozen hydrogen (*n*_H2_ = 1.14) (*34*), a tracer typically used in superfluid ^4^He. This high-refractive index allows the light to be scattered with high efficiency, leading to a larger signal-to-noise ratio and higher-speed observation. For the same nanoparticles size, the scattering cross section of silicon nanoparticles (*35*) was larger than that of solid hydrogen by two orders of magnitude (see the Supplementary Materials). The higher scattering efficiency also allowed us to use smaller particles as the quantized vortex tracer, ensuring a more passive role for the tracer. The size of the silicon nanoparticles used in this study was smaller than that of previously reported solid hydrogen [a few micrometer scales (*8*)] and solid atmospheric air [400 nm (*34*)]. The flexibility of the laser ablation technique allows us to control the nanoparticle size by changing laser ablation parameters, such as pulse duration, wavelength, and pulse energy (*36*). Various material options and size controllability are substantial advantages that provide an alternative method not only for quantized vortex visualization but also for liquid helium flow visualization. Moreover, these features are important for the incorporation of optical manipulation techniques (*19*, *20*) into quantum fluid research. Because of the high-refractive index, the polarizability of the silicon nanoparticles is an order of magnitude larger than that of the hydrogen particles. As polarizability represents the strength of the light matter interaction, the large polarizability opens the new possibilities for studying superfluid helium properties optically. One interesting example is the optical trapping and manipulation of quantized vortices, where the tracer particles can be used as an optical force mediator (*20*).

## MATERIALS AND METHODS

### Silicon nanoparticle injection and quantized vortex visualization in superfluid ^4^He

We placed a 3 cm–by–3 cm–by–3 cm cuvette in superfluid ^4^He. The entire experimental process was performed in this cuvette filled with the superfluid ^4^He. We mounted a single crystalline silicon target [Nilaco, n type, (111)] in the cuvette. The liquid helium temperature was maintained at approximately 1.4 K throughout the experiment. Nanosecond light pulses from a frequency-tripled Q-switched Nd:YAG laser (Quanta-Ray; wavelength, 355 nm; pulse duration, 10 ns; repetition rate, 10 Hz; pulse energy, 1 mJ) were focused onto the target surface with a spot size of ∼40 μm using a plano-convex lens with 200-mm focal length. The cuvette has a fused silica window for the pulsed laser light incidence. The pulse energy was chosen not to damage the window. We can observe the bright plume indicating the laser ablation. After approximately 200 laser shots, the ablation laser light was blocked, and the observation was started. The produced silicon nanoparticles were illuminated using a continuous-wave laser (Spectraphysics; wavelength, 532 nm; power, 1 W). The illuminating light was in the form of a laser sheet (full width, 300 μm), prepared using a set of cylindrical lenses. The effective numerical aperture of the imaging system (edmund optics, 54-691) is ∼0.021, and the overall magnification is ∼0.18. The spatial resolution of our observation system (19 μm) was primarily limited by the complementary metal-oxide semiconductor (CMOS) camera pixel size. All data were collected at 30 frames/s. The laser ablation and observation processes need to be repeated to record a sufficient number of reconnection events. The images for the quantized vortex observation were intensity-inverted and background-subtracted. Then, the contrast was enhanced to clarify the observed structures.

### SEM observation

The silicon nanoparticles fabricated via laser ablation are collected on a substrate at the bottom of the cuvette in the superfluid helium cryostat. The substrate was observed via SEM (JEOL, JSM-6500F) at room temperature. Randomly chosen areas are used to evaluate the size distribution of the fabricated nanoparticles. The magnification ranges from 3300× to 70,000× to precisely measure the particle dimensions. One typical SEM image is shown in the Fig. 1.

### Vortex filament calculations

Superfluid ^4^He can be described by a two-fluid model, i.e., it comprises an inviscid superfluid component and a viscous normal fluid component (*37*, *38*). Any rotational motion in superfluid flow is sustained by quantized vortex filaments (*39*–*41*). According to Helmholtz’s theorems, a vortex filament **s**(ξ, *t*) travels with a superfluid velocity **v**_s0_(ξ, *t*) induced at its location, where ξ is its arc length. The velocity can be found by calculating a Biot-Savart integral of the following form (*40*)

$$v_{s0}(r)=\frac{\kappa }{4\pi }\int_{L}\frac{s\prime (\xi )\times (s(\xi )-r)}{{|s(\xi )-r|}^{3}}d\xi$$ (3)

where the prime symbol represents a derivative with respect to ξ and ℒ is the path that coincides with the vortex filament. However, at finite temperature, the motion of a vortex line is modified because of the interactions with thermal excitations, such as phonons and rotons, comprising the normal fluid component. The effect of these interactions manifests as the drag **f**_D_ and the Magnus **f**_M_ forces acting of the vortex core per unit length. More explicitly

$$f_{D}=-\alpha \rho_{s}\kappa s\prime \times [s\prime \times (v_{n}-v_{s})]-\alpha \prime \rho_{s}\kappa s\prime \times (v_{n}-v_{s})$$ (4)

$$f_{M}=\rho_{s}\kappa s\prime \times (v_{L}-v_{s})$$ (5)

where **v**_L_ is the vortex core velocity and α and α′ are temperature-dependent friction coefficients. Assuming that the hydrodynamic effective mass of a vortex core per unit length is *m*_eff_, an equation of motion for a vortex core per unit length can be obtained. By assuming that *m*_eff_ is negligibly small, as the order of the vortex core radius is of 1Å, one can further simplify the equation of motion as follows

$$\frac{ds(\xi ,t)}{dt}=v_{L}=v_{s0}(\xi ,t)+\alpha s\prime \times (v_{n}-v_{s})-\alpha \prime s\prime \times [s\prime \times (v_{n}-v_{s})]$$ (6)

where **v***_n_* is some background normal fluid flow. The position of a filament **s**(ξ) was discretized and stored as a set of points separated by some resolution Δξ. Computationally, Δξ was set to be within a range of Δξ_min_ = 0.05 mm to Δξ_max_ = 0.1 mm, and the temporal resolution Δ*t* is 0.01 s. Then, we solved the integrodifferential equation (Eq. 6) adopting a fourth-order Runge-Kutta integration method for the temporal evolution.

Vortex reconnection events can be handled algorithmically in the VFM. When two vortices were within Δξ_min_ of each other, we exchanged the legs of the vortices. This method may seem arbitrary; however, we were primarily interested in the postreconnection dynamical scaling behavior in vortices, and the algorithm detail does not substantially affect the scaling law.

## Supplementary Materials

### This PDF file includes:

Scattering efficiency for various nanoparticles Motion of a particle bound to a vortex filament Fig. S1
