# Supplementary material for: Visualization of quantized vortex reconnection enabled by laser ablation
Source: Sci Adv. 2022 May 4;8(18):eabn1143. doi: 10.1126/sciadv.abn1143 (PMC9067918; doi:10.1126/sciadv.abn1143)
Supplement: Supplementary file 1 — Scattering efficiency for various nanoparticles Motion of a particle bound to a vortex filament Fig. S1 [file sciadv.abn1143_sm.pdf]

Science Advances
AAAS

Supplementary Materials for

Visualization of quantized vortex reconnection enabled by laser ablation

Yosuke Minowa*, Shota Aoyagi, Sosuke Inui, Tomo Nakagawa, Gamu Asaka,
Makoto Tsubota, Masaaki Ashida

*Corresponding author. Email: minowa@mp.es.osaka-u.ac.jp

Published 4 May 2022, *Sci. Adv.* **8**, eabn1143 (2022)
DOI: 10.1126/sciadv.abn1143

The PDF file includes:

Scattering efficiency for various nanoparticles
Motion of a particle bound to a vortex filament
Fig. S1
Legends for movies S1 to S3

Other Supplementary Material for this manuscript includes the following:

Movies S1 to S3

Scattering efficiency for various nanoparticles

Particle light scattering efficiency is determined using the refractive index and particle size. Under our experimental conditions, the particle size was smaller than the wavelength (532 nm) corresponding to the Rayleigh scattering. According to the Rayleigh scattering theory, the total scattering cross section can be written as

$$C_{\mathrm{sca}} = \frac{2\pi^5}{3}\frac{d^6}{\lambda^4}\left(\frac{n^2-1}{n^2+2}\right)^2$$

where d is the particle size, λ is the wavelength of the illuminating light, and n is the relative refractive index of the particles. According to the Rayleigh scattering theory, the light scattering angular distribution is exactly the same as the dipole radiation, and thus is not dependent on the material parameters of the nanoparticles. A comparison of the scattering efficiency of the silicon and solid hydrogen nanoparticles revealed the former to be larger than the latter by approximately two orders of magnitudes. When the particle size was approaching the wavelength of the illuminating light, the actual scattering began to deviate from the above-described Rayleigh approximation. We calculated the scattering cross section as a function of the particle size and the refractive index by applying the Mie scattering theory *(34)*. By assuming the particles to have a spherical shape, the total scattering cross section of nanoparticles is calculated as in Fig. S1. The total scattering cross section of the larger silicon nanoparticles yielded Mie resonance features for $d \gtrsim 100\,\mathrm{nm}$. Thus, nanoparticles of a certain size range tend to have a relatively higher scattering efficiency, although a slight size change could lead to the light scattering efficiency suppression. The steep wavelength dependence reflects the resonant nature of Mie scattering.

Motion of a particle bound to a vortex filament

First, consider the motion of a particle of density ρ_p and volume V_p (radius a_p) immersed in a classical fluid of density ρ_l. Ignoring the gravitational and buoyant forces, the equation of motion of such particle is given by

$$M_{eff}\boldsymbol{a} = \boldsymbol{F}_I + \boldsymbol{F}_S + \boldsymbol{F}_{ext}, \tag{1}$$

* minowa@mp.es.osaka-u.ac.jp

where M_{eff} is a hydrodynamic effective mass defined as $M_{eff} = (\rho_p + \rho_l/2)V_p$, $\boldsymbol{F}_I = \frac{3}{2}\rho_l V_p \frac{D\boldsymbol{v}}{Dt}$ is the inertial force owing to the acceleration of the background fluid, F_S is the Stokes drag force on the particle proportional to the difference between the particle and fluid velocities, and F_{ext} is some external force acting on the particle. The operation $\frac{D}{Dt}$ in the inertial force represents the material derivative of the fluid flow at the particle centre in the absence of the particle.

For the case of superfluid ^{4}He, we could extend this view straightforwardly, although we need to pay special attention to the complexity arising from its two-fluid nature. Unlike classical one-component fluids, superfluid ^{4}He has two independent fluid components, which requires us to rewrite the first term in left-hand side of Eq. (1) as

$$\boldsymbol{F}_I = \frac{3}{2}\rho_s V_p \frac{D\boldsymbol{v}_s}{Dt} + \frac{3}{2}\rho_n V_p \frac{D\boldsymbol{v}_n}{Dt}, \tag{2}$$

where subscripts, s and n represent super and normal components, respectively. The superfluid component is inviscid, so it does not contribute to F_S; however, the normal component possesses a finite viscosity μ_n and does contribute to this term, and thus

$$\boldsymbol{F}_S = 6\pi a_p \mu_n (\boldsymbol{v}_n - \boldsymbol{v}_p), \tag{3}$$

where $\boldsymbol{v}_p$ is the particle velocity. Next, with the presence of a quantized vortex, we would like to approximate the external force F_{ext} acting on the particle. If we assume that the particle size is negligibly small compared to the length of the vortex filament, the force acting on a small vortex filament segment is approximately equal to that acting on the particle, *i.e.* $\boldsymbol{F}_{ext} \approx \boldsymbol{F}_M + \boldsymbol{F}_D$, where the Magnus F_M and the drag F_D forces arise from the microscopical interactions between the vortex core and thermal excitations comprising the normal component. The leading order terms of these forces per unit length are known to be expressed as follows

$$\frac{\boldsymbol{F}_M}{\Delta\xi} \approx \rho_s \kappa \boldsymbol{s}' \times (\boldsymbol{v}_p - \boldsymbol{v}_s) \quad \text{and} \quad \frac{\boldsymbol{F}_D}{\Delta\xi} \approx \gamma_0 (\boldsymbol{v}_p - \boldsymbol{v}_n) \tag{4}$$

where γ_0 is the temperature-dependent friction coefficient.

Next, we would like to estimate the order of the forces to investigate the physical significance of each term. In the experiment, the particle size order is $a_p \sim \Delta\xi \sim 10^{-7}$ m and $M_{eff} \sim 10^{-18}$ kg. Once we assume the typical radius of curvature of a vortex filament is of 1 mm (see, for example, Fig. 2), then the typical vortex velocity is 10^{-4} m/s. From these

estimates, and using the values at $T = 1.4$ K from Refs.*(36, 37)*, we obtain the orders as follows

$$F_I \sim 10^{-23}\ \mathrm{N}, \quad F_S \sim 10^{-16}\ \mathrm{N}, \quad F_D \sim 10^{-17}\ \mathrm{N}, \quad F_M \sim 10^{-16}\ \mathrm{N}, \tag{5}$$

and if the particle moves with the vortex core cohesively,

$$M_{eff} a \sim 10^{-22}\ \mathrm{N}. \tag{6}$$

Because the inertial terms ($M_{eff}a$ and F_I) are smaller than other terms by several orders of magnitude, their effects may be negligible. Conversely, the dynamics of a vortex filament may be altered locally owing to the attached particle, as F_S is comparable to the other forces in typical situations. However, it should be emphasized that this modification should not affect the scaling exponent α in equation (1) in the main text because this is essentially related to the local structure of the vortex core characterized by its curvature. Nevertheless, the inertial terms can be a factor that modifies the scaling exponent. A vortex filament (and attached particles nearby) undergoes a large acceleration at the moment of reconnection, so for some short moment those terms (that are proportional to the acceleration) are not necessarily negligible. The acceleration a of a vortex at reconnection may be understood as the second derivative of distance d in equation (1) in the main text with respect to time:

$$a = -(A\sqrt{\kappa}/2)t^{-3/2}, \tag{7}$$

where $t = 0$ indicates the time when reconnection takes place. This implies that $M_{eff}\boldsymbol{a}$ is greater or similar to other forces for times shorter than $t \sim 1$ ms, and thus, the scaling behaviour could be broken below that time scale. However, this effect may not be resolved because the experimental observation is made with a 30-fps video recording.

Movie S1.

Quantised vortex motion visualized with silicon nanoparticles. Scale bars, 1 mm. The movie is played in real-time.

Movie S2.

Quantised vortex motion visualized with silicon nanoparticles. Scale bars, 1 mm. The movie is played in real-time.

Movie S3.

Reconnecting quantized vortices. Scale bars, 1 mm. The movie is played in real-time.

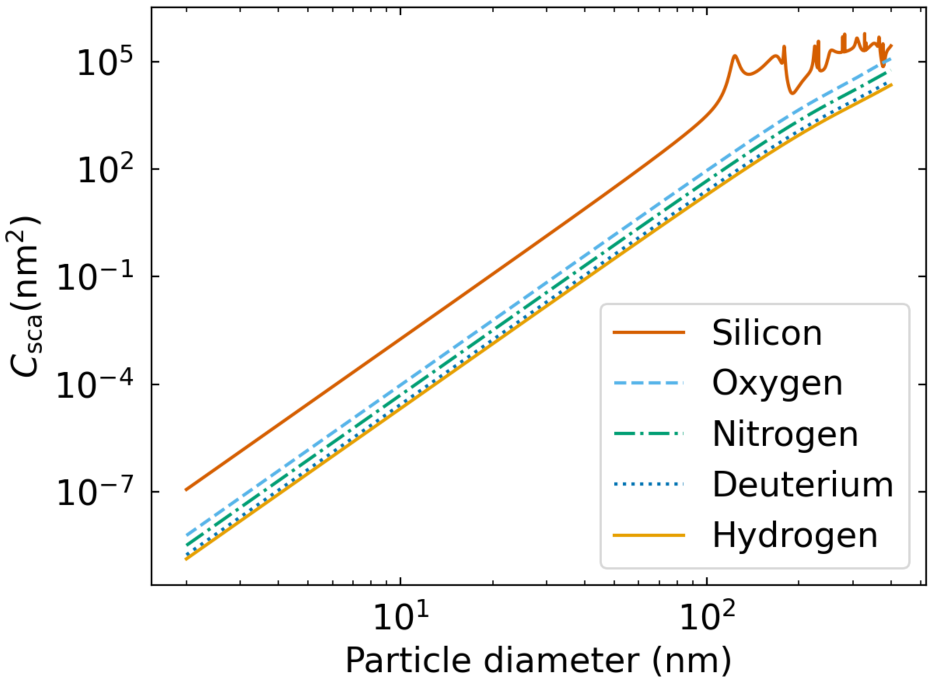

FIG. S1. **Total scattering cross section.** Particle size dependence of the total scattering cross section for different materials. A spherical shape is assumed.
